# Membranous nephropathy succeeding autologous hematopoietic stem cell transplant: a case report

**DOI:** 10.1186/s12882-018-0855-z

**Published:** 2018-03-09

**Authors:** Sanda Mrabet, Narjess Ben Aicha, Nihed Abdessayed, Moncef Mokni, Abdellatif Achour

**Affiliations:** 1 Department of Nephrology, Dialysis and transplantation. Sahloul university Hospital, Sousse, Tunisia; 2 Department of Pathology, Farhat Hached University Hospital, Sousse, Tunisia

**Keywords:** Membranous nephropathy, Glomerulonephritis, Autologous hematopoietic stem cell transplantation, Graft-vs-host disease

## Abstract

**Background:**

Membranous nephropathy (MN), the leading cause of nephrotic syndrome in adults, is characterized by the deposition of subepithelial immune deposits. Most of the cases are primary, while only approximately 25% of the cases are secondary to some known diseases. Recently, MN has been considered to be a possible presentation of chronic graft-versus-host disease (GVHD) of the kidney in allogeneic hematopoietic stem cell transplantation (HSCT) patients. In autologous HSCT populations, there have been scarce reports of associated MN, as a result of immune dysregulation leading to systemic autoimmunity and miming chronic GVHD.

**Case presentation:**

We report an exceptional case of MN associated to an acute renal failure occurring within days following an autologous HSCT indicated by multiple myeloma. There was no evidence of GVHD or myeloma relapse. A complete remission of nephrotic syndrome with normalization of renal function were rapidly obtained by corticosteroid therapy.

**Conclusion:**

This is the first published case of acute renal failure due to MN occurring in the acute phase of an autologous HSCT. These findings support the antibodymediated autoimmune glomerular disease.

## Background

Acute and even chronic renal injury are known complications of the Hematopoietc Stem Cell Transplant (HSCT) [1, 2]. They are attributed to overlapping causes such as tumor lysis syndrome, ischemic tubular necrosis, hemolytic uremic syndrome, infections and drugs induced nephrotoxicity [2, 3].

Glomerulopathies represent only a small proportion of such injury occuring in 1 to 6% of allogenic HSCT recipients [4–6]. They typically manifest as membranous nephropathy (MM) (64%), less commonly as minimal change disease (19%) and rarely as proliferative glomerulonephritis (17%) [7]. There is evidence to suggest that these glomerulopathies might represent manifestations of graft-versus-host disease (GVHD) [8].

With the emergence of autologous HSCT, GVHD should not exist. Nevertheless, Autologous GVHD has been described in up to 10% of patients after autologous HSCT [9], with involvement of skin, gastrointestinal tract or liver [10].

Recently, there have been scarce reports of associated MN in autologous HSCT population [11]. Some authors have questioned the possibility of its integration into GVHD while others considered it as a result of immune dysregulation leading to a systemic autoimmunity miming chronic GVHD. We describe the first published case of acute renal failure due to MN occurring in the acute phase of an autologous HSCT and we will try to find an explanation for its occurrence.

## Case presentation

A 54-year-old male with history of hypertension for 2 years was investigated for normochromic normocytic anemia in September 2013. Initial Laboratory data were: leukocyte count 6700/mm^3^, hemoglobin 9.7 g/dl, platelet count 226,000/mm^3^ and prothrombin time 32 s (79%). Erythrocyte sedimentation rate was 139/145,serum total protein was 7, 8 g/dl (including 31% γ-globulin with a monoclonal peak), Immunophenotyping: peak monoclonal IgG Kappa, albumin 3,8 g/dl, creatinine 78 μmol/l, blood urea nitrogen 7 mmol/l, calcemia 2,2 mmol/l, blood uric acid 532 μmol/l. The 24-h urinary excretion of total protein was 1 g/d with Bence-Jones proteinuria of 0, 6 g. Bone marrow aspiration showed myeloma cell infiltration in the bone marrow (31% of dystrophic plasma cells). The diagnosis of IgG Kappa multiple myeloma was made and he began, in December 2013, his induction treatment with Thalidomide-Dexamethasone receiving 3 cycles with good response. Then, he underwent in June 2014 autologous SCT with high-dose melphalan.

**Fig. 1:**
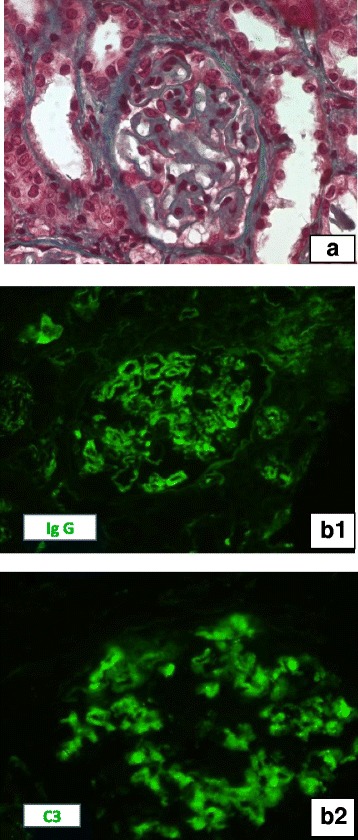
Kidney biopsy Light microscopy (A): A glomerulus with important thickening of glomerular capillary wall and normal cellularity(Masson Trichrom, × 400). Immunofluorescence studies: Intense granular staining of IgG (B1) and C3 (B2) along the glomerular capillaries (direct immunofluorescence, × 400)

During the second week, Autograft was complicated by a urinary tract infection and sepsis Staphylococcus hemolyticus with development of acute renal failure, the blood creatinine level reaching 336 μmol/l. Renal function improved on antibiotics but has not normalized so the patient was referred to Nephrology when his blood count was normalized for further evaluation of his renal failure. At Nephrology (day 29), the blood creatinine level was 260 μmol/l and multiple myeloma was in total remission. However, there was a progressive increase in proteinuria becoming nephrotic. Bence- Jones proteinuria remained negative. A kidney biopsy was then performed showing features of membranous nephropathy (Fig. 1). No secondary causes were present to explain the MN. He had undergone testing for hepatitis, syphilis, anti-thyroid antibodies, colonoscopy, PSA screen, CT scan of lung, abdomen and pelvis, all of which were negative. In addition, the patient’s serum was tested for phospholipase A2 receptor (PLA2R) autoantibodies, which were also negative.

The patient was put under renin-angiotensin system blockers. Renal function improved (creatinine 160 μmol / l after 3 months), the proteinuria remained oscillating between 3,5 and 11 g / l. After one year of monitoring, diagnosis was discussed again on the basis of a recent bibliography and a corticoid treatment, prednisone at a dose of 1 mg / kg / day was introduced targeting an autoimmune process. The outcome was favorable with a decrease of proteinuria remaining between 0.6 and 0.8 g / d and a creatinine down to 103 μmol/l at 3 months of treatment. Corticosteroid degression was started after 4 months motivated by the good evolution and development of cortico-induced diabetes. Total duration of corticotherapy was 8 months. At last check (in February 2017, one year after starting corticotherapy), renal function was stable (creatinine at 102 μmol/l), PLA2R autoantibodies and proteinuria were negative.

## Discussion

GVHD is a potentially lethal condition in which transplanted donor leukocytes (graft) attack the recipient’s tissue (host) [12]. GVHD often occurs after HSCT and may be acute or chronic. Acute GVHD presents within 100 days of transplantation and typically affects the skin, gastrointestinal tract and liver [13]. Chronic GVHD occurs more than 100 days posttransplantation, affects multiple organs (skin, eyes, mouth, esophagus, liver, etc) and often is associated with autoimmune features [13]. Chronic GVHD can occur after a history of acute GVHD or as a de novo phenomenon.

Post-allogeneic HSCT glomerulopathies most often manifest as nephrotic syndrome caused primarily by MN [7]. GVHD is believed to be the cause of postallogeneic HSCT secondary MN [8] since the majority of patients with post-HSCT MN had a history of acute (68%) or chronic (75% to 84%) GVHD [7, 8, 14] and since there is an increased incidence of post-HSCT MN compared to the general population (0.6%–3.8% over 1–10 years in post-HSCT patients [7] vs an annual incidence of 0.0012% in the general population [15].

With the emergence of autologous HSCT, GVHD and its complications, quite common in allogeneic transplantation,were expected to be eliminated. However, autologous GVHD has been described in some patients after autologous hematopoietic HSCT.

Recent studies have indicated that two major factors are necessary for the induction of autologous GVHD. The first factor was a disruption of thymic-dependent immune reconstitution and the second one was a failure to re-establish peripheral self-tolerance [8].

Once the alloreactive T cells are released to the periphery, they can be eliminated using a T-cell-dependent regulatory system, however, due to the lymphoablative preparative regimen that HSCT patients undergo, this system is not functional [7, 16].

On another hand, autologous GVHD has been thought of as an autoimmune syndrome and a milder form of GVHD than its counterpart in allogeneic transplantation. In recent studies, the presence of auto-antibodies in relation to the HSCT provides evidence for the involvement of the B cell. A possible theory on how B cells can contribute to chronic GVHD is that the reconstituted B cells after myeloablative conditioning may have impaired immune tolerance of peripheral B cells, leading to the production of autoantibody in chronic GVHD [17].

In autologous HSCT populations, there have been reports of associated glomerular disease, as a result of any type of immune dysregulation. Based on literature, 6% of all patients with glomerulonephritis after HCT had received Autologous transplants [7, 18, 19].

MN is encountered very rarely following autologous HSCT (3% postautologous vs 97% postallogeneic HSCT [7]. In this context of autoimmunity lymphokines would alter the podocytes causing MN. New factors appearing after the HCT could also play a role.

Nephrotic syndrome was described to be a late event after HSCT either post alloneneic HSCT or autologous HSCT. Indeed, nephrotic syndrome occurring in allogeneic HSCT patients has been considered to be a possible presentation of chronic GVHD of the kidney and a decrease in immunosuppressive medication after HSCT was associated with the occurrence of nephrotic syndrome within 9 months in 63% of patients [7, 20]. In the last report of case of MN after autologous HSCT, proteinuria appeared 5 years after HSCT.

Here we presented a case of a MN diagnosed few days after a HSCT without features of acute GVHD. Primary MN was initially evoked, however the absence of anti-PLA2R antibody rendered the possibility of the coincidental development of primary MN less likely. More, our patient showed complete remission to steroid treatment which is in favor of post-HSCT MN. Indeed, as opposed to primary MN, corticosteroids, usually are the first line of treatment for post-HSCT MN [6, 14, 21].

## Conclusion

Although post HSCT MN likely represents a late manifestation of chronic GVHD in allogeneic HSCT, it may also occur after autologous HSCT. Auto immunity seems to be on the basis of its occurrence as supported by the glomerular deposition of IgG and complement components. Auto immunity could be acute and involve only the kidneys as seen in our case. Corticosteroids are the principal treatment toil and may be sufficient.

Through this observation, we recommend more screening protocols of proteinuria and renal function post HSCT.
